# A new ortho­rhom­bic polymorph of 1,1′-seleno­bis­(*N*,*N*-diethyl­sulfanecarbothio­amide)

**DOI:** 10.1107/S1600536811000511

**Published:** 2011-01-15

**Authors:** Alpar Pöllnitz, Richard A. Varga, Anca Silvestru

**Affiliations:** a Faculty of Chemistry and Chemical Engineering, Babes-Bolyai University, 11 Arany Janos St., RO-400028, Cluj Napoca, Romania

## Abstract

The title compound [systematic name: *N*,*N*-dieth­yl({[(diethyl­carbamothio­yl)sulfan­yl]selan­yl}sulfan­yl)carbothio­amide], C_10_H_20_N_2_S_4_Se, crystallizes in a new form in the space group *Pca*2_1_: the previously reported polymorph crystallizes in the space group *P*2_1_2_1_2_1_. The new phase contains two independent mol­ecules in the asymmetric unit. The Se atoms are tetra­coordinated, with a distorted square-planar geometry. The ligands coordinate asymmetrically to the Se atoms, with one strong Se—S bond [range 2.2833 (13)–2.3041 (15) Å] and one weaker bond [range 2.7318 (14)–2.7873 (12) Å].

## Related literature

For the characterization of the *P*2_1_2_1_2_1_ polymorph, see: Conde *et al.* (1970 ▶); Husebye & Helland-Madsen (1970 ▶); Sugihara (1985 ▶). For the benzene solvate, see: Klapötke *et al.* (2008 ▶). For the synthesis of bis­(*o*-formyl­phen­yl)selenide acetal, see: Panda *et al.* (2001 ▶).
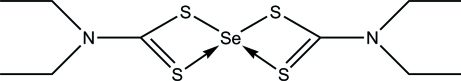


## Experimental

### 

#### Crystal data

C_10_H_20_N_2_S_4_Se
*M* *_r_* = 375.52
Orthorhombic, 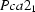

*a* = 16.0338 (18) Å
*b* = 15.6376 (18) Å
*c* = 13.5880 (15) Å
*V* = 3406.9 (7) Å^3^
*Z* = 8
Mo *K*α radiation
μ = 2.68 mm^−1^
*T* = 297 K
0.27 × 0.26 × 0.19 mm

#### Data collection

Bruker SMART APEX diffractometer
Absorption correction: multi-scan (*SADABS*; Bruker, 2001 ▶) *T*_min_ = 0.532, *T*_max_ = 0.630
17395 measured reflections
5896 independent reflections
4856 reflections with *I* > 2σ(*I*)
*R*_int_ = 0.046

#### Refinement

*R*[*F* ^2^ > 2σ(*F* ^2^)] = 0.038
*wR*(*F* ^2^) = 0.076
*S* = 0.94
5896 reflections
315 parameters
1 restraint
H-atom parameters constrained
Δρ_max_ = 0.36 e Å^−3^
Δρ_min_ = −0.22 e Å^−3^
Absolute structure: Flack (1983 ▶), 2755 Friedel pairs
Flack parameter: 0.007 (7)

### 

Data collection: *SMART* (Bruker, 2001 ▶); cell refinement: *SAINT-Plus* (Bruker, 2001 ▶); data reduction: *SAINT-Plus*; program(s) used to solve structure: *SHELXS97* (Sheldrick, 2008 ▶); program(s) used to refine structure: *SHELXL97* (Sheldrick, 2008 ▶); molecular graphics: *ORTEP-3* (Farrugia, 1997 ▶); software used to prepare material for publication: *publCIF* (Westrip, 2010 ▶).

## Supplementary Material

Crystal structure: contains datablocks I, global. DOI: 10.1107/S1600536811000511/bh2326sup1.cif

Structure factors: contains datablocks I. DOI: 10.1107/S1600536811000511/bh2326Isup2.hkl

Additional supplementary materials:  crystallographic information; 3D view; checkCIF report

## Figures and Tables

**Fig. 1.**
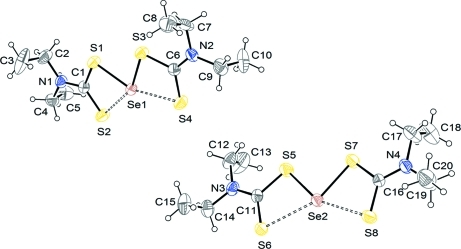
The molecular structure of the title compound, showing 30% probability displacement ellipsoids and the atom-numbering scheme.

## supplementary crystallographic information

### Comment

In the crystal, the title compound contains two independent molecules in the asymmetric unit, with only slight differences in the bond distances and bond angles between the two molecules (Fig. 1). Both Se atoms are tetracoordinated with four S atoms arranged in a distorted square-planar geometry. The distortion of the coordination geometry is the result of the small bite and asymmetric coordination of the ligands. Each ligand coordinates strongly to the corresponding Se center through one S atom [range 2.2833 (13)–2.3041 (15) Å] and forms a weaker interaction through the second S atom [range 2.7318 (14)–2.7873 (12) Å].

The molecular structure is similar to the ones published (Klapötke *et al.*, 2008; Sugihara, 1985; Husebye & Helland-Madsen, 1970; Conde *et al.*, 1970) in terms of structure parameters. Only one difference can be observed between the title compound and the benzene solvate (Klapötke *et al.*, 2008), the position of the Et groups from the ligands. While in the title compound the organic fragments from the same ligand are on the opposite sides of the coordination plane, in the benzene solvate they are on the same side.

### Experimental

The title compound was recovered as unreacted material after the reaction described for the synthesis of bis(*o*-formylphenyl)selenide acetal by Panda *et al.* (2001). Recrystallization from diethylether at -20°C over 72 h afforded suitable crystals.

### Refinement

All hydrogen atoms were placed in calculated positions using a riding model, with C—H = 0.96–0.97 Å and with *U*_iso_= 1.2 or 1.5*U*_eq_ (C) for H. The methyl groups were allowed to rotate but not to tip.

### Crystal data

**Table:**

C_10_H_20_N_2_S_4_Se	*F*(000) = 1536
*M**_r_* = 375.52	*D*_x_ = 1.464 Mg m^−^^3^
Orthorhombic, *P**c**a*2_1_	Mo *K*α radiation, λ = 0.71073 Å
Hall symbol: P 2c -2ac	Cell parameters from 3414 reflections
*a* = 16.0338 (18) Å	θ = 2.4–21.5°
*b* = 15.6376 (18) Å	µ = 2.68 mm^−^^1^
*c* = 13.5880 (15) Å	*T* = 297 K
*V* = 3406.9 (7) Å^3^	Block, orange
*Z* = 8	0.27 × 0.26 × 0.19 mm

### Data collection

**Table:**

Bruker SMART APEX diffractometer	5896 independent reflections
Radiation source: fine-focus sealed tube	4856 reflections with *I* > 2σ(*I*)
graphite	*R*_int_ = 0.046
φ and ω scans	θ_max_ = 25.0°, θ_min_ = 2.4°
Absorption correction: multi-scan (*SADABS*; Bruker, 2001)	*h* = −18→19
*T*_min_ = 0.532, *T*_max_ = 0.630	*k* = −18→17
17395 measured reflections	*l* = −16→15

### Refinement

**Table:**

Refinement on *F*^2^	Secondary atom site location: difference Fourier map
Least-squares matrix: full	Hydrogen site location: inferred from neighbouring sites
*R*[*F*^2^ > 2σ(*F*^2^)] = 0.038	H-atom parameters constrained
*wR*(*F*^2^) = 0.076	*w* = 1/[σ^2^(*F*_o_^2^)] where *P* = (*F*_o_^2^ + 2*F*_c_^2^)/3
*S* = 0.94	(Δ/σ)_max_ = 0.001
5896 reflections	Δρ_max_ = 0.36 e Å^−^^3^
315 parameters	Δρ_min_ = −0.22 e Å^−^^3^
1 restraint	Absolute structure: Flack (1983), 2755 Friedel pairs
0 constraints	Flack parameter: 0.007 (7)
Primary atom site location: structure-invariant direct methods	

### Fractional atomic coordinates and isotropic or equivalent isotropic displacement parameters (Å^2^)

**Table:**

	*x*	*y*	*z*	*U*_iso_*/*U*_eq_	
C1	0.2875 (3)	0.5449 (3)	0.9917 (3)	0.0528 (11)	
C2	0.2975 (4)	0.4729 (4)	1.1515 (5)	0.0899 (18)	
H2A	0.2578	0.4655	1.2046	0.108*	
H2B	0.3357	0.5184	1.1697	0.108*	
C3	0.3460 (5)	0.3900 (6)	1.1355 (9)	0.172 (4)	
H3A	0.3078	0.3448	1.1194	0.258*	
H3B	0.3757	0.3756	1.1946	0.258*	
H3C	0.3849	0.3976	1.0825	0.258*	
C4	0.1673 (3)	0.4642 (4)	1.0520 (4)	0.0766 (16)	
H4A	0.1626	0.4094	1.0849	0.092*	
H4B	0.1542	0.4558	0.9830	0.092*	
C5	0.1059 (3)	0.5257 (5)	1.0961 (5)	0.106 (2)	
H5A	0.1192	0.5346	1.1642	0.159*	
H5B	0.0506	0.5025	1.0907	0.159*	
H5C	0.1086	0.5792	1.0616	0.159*	
C6	0.5408 (3)	0.7480 (3)	0.7986 (4)	0.0540 (12)	
C7	0.6744 (3)	0.7964 (4)	0.8659 (5)	0.0879 (18)	
H7A	0.6486	0.7904	0.9301	0.105*	
H7B	0.7018	0.8517	0.8639	0.105*	
C8	0.7384 (4)	0.7283 (4)	0.8539 (6)	0.104 (2)	
H8A	0.7117	0.6734	0.8543	0.156*	
H8B	0.7777	0.7314	0.9071	0.156*	
H8C	0.7671	0.7362	0.7925	0.156*	
C9	0.6213 (4)	0.8503 (4)	0.7051 (5)	0.0860 (18)	
H9A	0.5988	0.8232	0.6466	0.103*	
H9B	0.6806	0.8588	0.6949	0.103*	
C10	0.5794 (4)	0.9369 (4)	0.7195 (7)	0.130 (3)	
H10A	0.5209	0.9287	0.7309	0.195*	
H10B	0.5872	0.9711	0.6616	0.195*	
H10C	0.6038	0.9653	0.7751	0.195*	
C11	0.5475 (2)	0.7900 (3)	0.2665 (4)	0.0511 (11)	
C12	0.4976 (4)	0.7850 (4)	0.4376 (4)	0.0868 (18)	
H12A	0.5448	0.8218	0.4516	0.104*	
H12B	0.4982	0.7387	0.4850	0.104*	
C13	0.4186 (5)	0.8354 (5)	0.4500 (6)	0.136 (3)	
H13A	0.4187	0.8829	0.4053	0.205*	
H13B	0.4150	0.8561	0.5164	0.205*	
H13C	0.3715	0.7993	0.4364	0.205*	
C14	0.4681 (3)	0.6663 (3)	0.3214 (5)	0.0836 (18)	
H14A	0.4498	0.6628	0.2535	0.100*	
H14B	0.4193	0.6611	0.3632	0.100*	
C15	0.5265 (5)	0.5937 (4)	0.3426 (7)	0.127 (3)	
H15A	0.5748	0.5984	0.3011	0.190*	
H15B	0.4987	0.5405	0.3297	0.190*	
H15C	0.5433	0.5958	0.4103	0.190*	
C16	0.7525 (3)	1.0497 (3)	0.0838 (3)	0.0551 (12)	
C17	0.8189 (3)	1.1743 (4)	0.1579 (5)	0.093 (2)	
H17A	0.8760	1.1945	0.1537	0.112*	
H17B	0.8135	1.1422	0.2186	0.112*	
C18	0.7628 (4)	1.2492 (4)	0.1626 (7)	0.127 (3)	
H18A	0.7674	1.2815	0.1028	0.191*	
H18B	0.7782	1.2846	0.2174	0.191*	
H18C	0.7063	1.2302	0.1707	0.191*	
C19	0.8443 (4)	1.1369 (4)	−0.0176 (5)	0.0847 (18)	
H19A	0.8535	1.1982	−0.0209	0.102*	
H19B	0.8079	1.1213	−0.0717	0.102*	
C20	0.9255 (4)	1.0925 (4)	−0.0301 (5)	0.105 (2)	
H20A	0.9640	1.1124	0.0188	0.158*	
H20B	0.9473	1.1043	−0.0945	0.158*	
H20C	0.9175	1.0320	−0.0228	0.158*	
N1	0.2536 (3)	0.4961 (2)	1.0614 (3)	0.0615 (11)	
N2	0.6087 (2)	0.7942 (2)	0.7896 (3)	0.0598 (10)	
N3	0.5070 (3)	0.7493 (3)	0.3382 (3)	0.0637 (11)	
N4	0.8028 (2)	1.1167 (3)	0.0749 (3)	0.0633 (11)	
S1	0.38904 (8)	0.58095 (9)	1.01289 (10)	0.0616 (3)	
S2	0.24068 (9)	0.57133 (9)	0.88656 (11)	0.0775 (4)	
S3	0.52830 (8)	0.68637 (9)	0.90454 (10)	0.0632 (3)	
S4	0.46334 (8)	0.74428 (10)	0.71483 (10)	0.0758 (4)	
S5	0.58959 (8)	0.88991 (8)	0.29503 (10)	0.0669 (4)	
S6	0.56063 (7)	0.75098 (8)	0.15308 (10)	0.0597 (3)	
S7	0.70132 (9)	1.03237 (8)	0.19479 (10)	0.0675 (4)	
S8	0.73695 (10)	0.97916 (9)	−0.00757 (10)	0.0794 (4)	
Se1	0.39627 (3)	0.64649 (3)	0.86143 (4)	0.05358 (13)	
Se2	0.64458 (3)	0.90662 (3)	0.13955 (4)	0.05516 (13)	

### Atomic displacement parameters (Å^2^)

**Table:**

	*U*^11^	*U*^22^	*U*^33^	*U*^12^	*U*^13^	*U*^23^
C1	0.053 (3)	0.051 (3)	0.055 (3)	0.006 (2)	0.009 (2)	0.002 (2)
C2	0.091 (4)	0.120 (5)	0.058 (4)	−0.010 (4)	−0.002 (4)	0.030 (4)
C3	0.183 (8)	0.161 (8)	0.171 (9)	0.073 (7)	0.025 (8)	0.103 (8)
C4	0.082 (4)	0.083 (4)	0.065 (4)	−0.028 (3)	0.004 (3)	0.002 (3)
C5	0.062 (4)	0.154 (7)	0.101 (5)	−0.020 (4)	0.015 (4)	−0.019 (5)
C6	0.061 (3)	0.052 (3)	0.049 (3)	0.004 (2)	0.010 (2)	−0.007 (2)
C7	0.094 (4)	0.098 (5)	0.071 (4)	−0.043 (4)	−0.007 (4)	−0.022 (4)
C8	0.068 (4)	0.120 (5)	0.123 (6)	−0.009 (4)	−0.018 (4)	0.013 (5)
C9	0.077 (4)	0.089 (5)	0.093 (5)	−0.022 (3)	0.020 (3)	0.005 (4)
C10	0.126 (6)	0.075 (5)	0.188 (9)	−0.001 (4)	0.034 (6)	0.053 (5)
C11	0.043 (2)	0.050 (3)	0.061 (3)	0.004 (2)	0.002 (2)	0.009 (2)
C12	0.108 (4)	0.098 (5)	0.055 (4)	0.016 (4)	0.023 (3)	0.017 (3)
C13	0.151 (6)	0.162 (8)	0.096 (6)	0.070 (6)	0.046 (5)	0.010 (5)
C14	0.083 (4)	0.076 (4)	0.092 (5)	−0.015 (3)	0.017 (3)	0.017 (3)
C15	0.170 (7)	0.070 (4)	0.141 (7)	0.010 (4)	−0.009 (6)	0.035 (5)
C16	0.063 (3)	0.045 (3)	0.057 (3)	−0.005 (2)	−0.004 (3)	0.002 (2)
C17	0.080 (4)	0.084 (4)	0.115 (6)	−0.024 (3)	0.012 (4)	−0.033 (4)
C18	0.088 (4)	0.090 (5)	0.204 (9)	−0.012 (4)	0.050 (5)	−0.042 (5)
C19	0.105 (5)	0.069 (4)	0.080 (4)	−0.035 (3)	0.016 (4)	0.006 (3)
C20	0.110 (5)	0.104 (5)	0.101 (5)	−0.028 (4)	0.036 (5)	−0.018 (4)
N1	0.063 (3)	0.064 (3)	0.058 (3)	−0.004 (2)	0.004 (2)	0.007 (2)
N2	0.062 (2)	0.054 (2)	0.064 (3)	−0.0117 (19)	0.010 (2)	−0.005 (2)
N3	0.072 (3)	0.066 (3)	0.053 (3)	0.001 (2)	0.013 (2)	0.009 (2)
N4	0.068 (3)	0.053 (2)	0.070 (3)	−0.014 (2)	0.003 (2)	−0.002 (2)
S1	0.0544 (7)	0.0774 (9)	0.0529 (8)	−0.0004 (6)	−0.0011 (6)	0.0125 (6)
S2	0.0656 (8)	0.1032 (10)	0.0637 (9)	−0.0092 (8)	−0.0120 (7)	0.0199 (8)
S3	0.0588 (7)	0.0774 (9)	0.0534 (8)	−0.0031 (6)	0.0000 (6)	0.0056 (7)
S4	0.0649 (8)	0.0990 (11)	0.0635 (10)	−0.0127 (7)	−0.0047 (7)	0.0243 (8)
S5	0.0789 (9)	0.0604 (8)	0.0614 (8)	−0.0046 (6)	0.0107 (7)	−0.0104 (7)
S6	0.0708 (7)	0.0561 (7)	0.0522 (7)	−0.0081 (6)	0.0056 (7)	−0.0016 (6)
S7	0.0809 (9)	0.0592 (8)	0.0624 (8)	−0.0170 (7)	0.0135 (7)	−0.0101 (7)
S8	0.1033 (10)	0.0782 (10)	0.0565 (9)	−0.0355 (8)	0.0075 (8)	−0.0147 (7)
Se1	0.0552 (3)	0.0547 (3)	0.0509 (3)	0.0034 (2)	0.0032 (3)	0.0038 (2)
Se2	0.0558 (3)	0.0524 (3)	0.0572 (3)	−0.0074 (2)	0.0025 (3)	−0.0011 (3)

### Geometric parameters (Å, °)

**Table:**

C1—N1	1.332 (6)	C12—C13	1.501 (8)
C1—S2	1.666 (5)	C12—H12A	0.9700
C1—S1	1.747 (5)	C12—H12B	0.9700
C2—N1	1.458 (7)	C13—H13A	0.9600
C2—C3	1.527 (10)	C13—H13B	0.9600
C2—H2A	0.9700	C13—H13C	0.9600
C2—H2B	0.9700	C14—N3	1.457 (7)
C3—H3A	0.9600	C14—C15	1.499 (8)
C3—H3B	0.9600	C14—H14A	0.9700
C3—H3C	0.9600	C14—H14B	0.9700
C4—N1	1.476 (6)	C15—H15A	0.9600
C4—C5	1.501 (8)	C15—H15B	0.9600
C4—H4A	0.9700	C15—H15C	0.9600
C4—H4B	0.9700	C16—N4	1.327 (6)
C5—H5A	0.9600	C16—S8	1.680 (5)
C5—H5B	0.9600	C16—S7	1.738 (5)
C5—H5C	0.9600	C17—N4	1.467 (7)
C6—N2	1.312 (5)	C17—C18	1.478 (8)
C6—S4	1.685 (5)	C17—H17A	0.9700
C6—S3	1.744 (5)	C17—H17B	0.9700
C7—N2	1.479 (7)	C18—H18A	0.9600
C7—C8	1.488 (8)	C18—H18B	0.9600
C7—H7A	0.9700	C18—H18C	0.9600
C7—H7B	0.9700	C19—N4	1.457 (7)
C8—H8A	0.9600	C19—C20	1.485 (8)
C8—H8B	0.9600	C19—H19A	0.9700
C8—H8C	0.9600	C19—H19B	0.9700
C9—N2	1.460 (7)	C20—H20A	0.9600
C9—C10	1.523 (8)	C20—H20B	0.9600
C9—H9A	0.9700	C20—H20C	0.9600
C9—H9B	0.9700	S1—Se1	2.3020 (14)
C10—H10A	0.9600	S3—Se1	2.2833 (13)
C10—H10B	0.9600	S5—Se2	2.3041 (15)
C10—H10C	0.9600	S7—Se2	2.2930 (13)
C11—N3	1.332 (6)	S8—Se2	2.7343 (15)
C11—S6	1.671 (5)	S6—Se2	2.7873 (12)
C11—S5	1.746 (5)	S2—Se1	2.7788 (15)
C12—N3	1.470 (7)	S4—Se1	2.7318 (14)
			
N1—C1—S2	124.6 (3)	C12—C13—H13A	109.5
N1—C1—S1	116.6 (3)	C12—C13—H13B	109.5
S2—C1—S1	118.7 (3)	H13A—C13—H13B	109.5
N1—C2—C3	109.7 (6)	C12—C13—H13C	109.5
N1—C2—H2A	109.7	H13A—C13—H13C	109.5
C3—C2—H2A	109.7	H13B—C13—H13C	109.5
N1—C2—H2B	109.7	N3—C14—C15	112.2 (5)
C3—C2—H2B	109.7	N3—C14—H14A	109.2
H2A—C2—H2B	108.2	C15—C14—H14A	109.2
C2—C3—H3A	109.5	N3—C14—H14B	109.2
C2—C3—H3B	109.5	C15—C14—H14B	109.2
H3A—C3—H3B	109.5	H14A—C14—H14B	107.9
C2—C3—H3C	109.5	C14—C15—H15A	109.5
H3A—C3—H3C	109.5	C14—C15—H15B	109.5
H3B—C3—H3C	109.5	H15A—C15—H15B	109.5
N1—C4—C5	111.3 (5)	C14—C15—H15C	109.5
N1—C4—H4A	109.4	H15A—C15—H15C	109.5
C5—C4—H4A	109.4	H15B—C15—H15C	109.5
N1—C4—H4B	109.4	N4—C16—S8	122.7 (4)
C5—C4—H4B	109.4	N4—C16—S7	119.3 (4)
H4A—C4—H4B	108.0	S8—C16—S7	118.0 (3)
C4—C5—H5A	109.5	N4—C17—C18	114.4 (6)
C4—C5—H5B	109.5	N4—C17—H17A	108.7
H5A—C5—H5B	109.5	C18—C17—H17A	108.7
C4—C5—H5C	109.5	N4—C17—H17B	108.7
H5A—C5—H5C	109.5	C18—C17—H17B	108.7
H5B—C5—H5C	109.5	H17A—C17—H17B	107.6
N2—C6—S4	124.6 (4)	C17—C18—H18A	109.5
N2—C6—S3	118.4 (4)	C17—C18—H18B	109.5
S4—C6—S3	117.0 (3)	H18A—C18—H18B	109.5
N2—C7—C8	113.4 (5)	C17—C18—H18C	109.5
N2—C7—H7A	108.9	H18A—C18—H18C	109.5
C8—C7—H7A	108.9	H18B—C18—H18C	109.5
N2—C7—H7B	108.9	N4—C19—C20	113.4 (6)
C8—C7—H7B	108.9	N4—C19—H19A	108.9
H7A—C7—H7B	107.7	C20—C19—H19A	108.9
C7—C8—H8A	109.5	N4—C19—H19B	108.9
C7—C8—H8B	109.5	C20—C19—H19B	108.9
H8A—C8—H8B	109.5	H19A—C19—H19B	107.7
C7—C8—H8C	109.5	C19—C20—H20A	109.5
H8A—C8—H8C	109.5	C19—C20—H20B	109.5
H8B—C8—H8C	109.5	H20A—C20—H20B	109.5
N2—C9—C10	111.8 (5)	C19—C20—H20C	109.5
N2—C9—H9A	109.2	H20A—C20—H20C	109.5
C10—C9—H9A	109.2	H20B—C20—H20C	109.5
N2—C9—H9B	109.2	C1—N1—C2	122.8 (4)
C10—C9—H9B	109.2	C1—N1—C4	121.0 (4)
H9A—C9—H9B	107.9	C2—N1—C4	116.2 (4)
C9—C10—H10A	109.5	C6—N2—C9	121.3 (5)
C9—C10—H10B	109.5	C6—N2—C7	122.6 (4)
H10A—C10—H10B	109.5	C9—N2—C7	116.0 (4)
C9—C10—H10C	109.5	C11—N3—C14	121.3 (4)
H10A—C10—H10C	109.5	C11—N3—C12	122.7 (4)
H10B—C10—H10C	109.5	C14—N3—C12	116.0 (5)
N3—C11—S6	124.1 (4)	C16—N4—C19	121.9 (4)
N3—C11—S5	117.0 (4)	C16—N4—C17	121.4 (5)
S6—C11—S5	118.9 (3)	C19—N4—C17	116.7 (4)
N3—C12—C13	112.9 (6)	C1—S1—Se1	92.48 (16)
N3—C12—H12A	109.0	C6—S3—Se1	92.62 (16)
C13—C12—H12A	109.0	C11—S5—Se2	92.63 (16)
N3—C12—H12B	109.0	C16—S7—Se2	92.13 (16)
C13—C12—H12B	109.0	S3—Se1—S1	86.50 (5)
H12A—C12—H12B	107.8	S7—Se2—S5	87.07 (5)
			
S2—C1—N1—C2	178.6 (4)	C13—C12—N3—C11	92.4 (7)
S1—C1—N1—C2	−0.1 (6)	C13—C12—N3—C14	−86.8 (7)
S2—C1—N1—C4	−3.0 (7)	S8—C16—N4—C19	4.1 (7)
S1—C1—N1—C4	178.4 (4)	S7—C16—N4—C19	−177.0 (4)
C3—C2—N1—C1	−89.6 (7)	S8—C16—N4—C17	−176.3 (4)
C3—C2—N1—C4	91.9 (6)	S7—C16—N4—C17	2.5 (7)
C5—C4—N1—C1	−89.9 (6)	C20—C19—N4—C16	−87.4 (6)
C5—C4—N1—C2	88.7 (6)	C20—C19—N4—C17	93.0 (6)
S4—C6—N2—C9	2.5 (7)	C18—C17—N4—C16	−92.6 (7)
S3—C6—N2—C9	−177.1 (4)	C18—C17—N4—C19	87.0 (7)
S4—C6—N2—C7	179.0 (4)	N1—C1—S1—Se1	176.8 (3)
S3—C6—N2—C7	−0.5 (6)	S2—C1—S1—Se1	−2.0 (3)
C10—C9—N2—C6	84.1 (7)	N2—C6—S3—Se1	174.3 (3)
C10—C9—N2—C7	−92.7 (6)	S4—C6—S3—Se1	−5.3 (3)
C8—C7—N2—C6	88.1 (6)	N3—C11—S5—Se2	179.0 (3)
C8—C7—N2—C9	−95.2 (6)	S6—C11—S5—Se2	−0.6 (3)
S6—C11—N3—C14	−1.7 (7)	N4—C16—S7—Se2	−176.1 (4)
S5—C11—N3—C14	178.8 (4)	S8—C16—S7—Se2	2.8 (3)
S6—C11—N3—C12	179.2 (4)	C6—S3—Se1—S1	−174.55 (15)
S5—C11—N3—C12	−0.4 (6)	C1—S1—Se1—S3	−179.80 (16)
C15—C14—N3—C11	90.1 (7)	C16—S7—Se2—S5	176.19 (17)
C15—C14—N3—C12	−90.7 (6)	C11—S5—Se2—S7	−176.75 (15)
